# Supplementary material for: State of the Art of Immune Checkpoint Inhibitors in Unresectable Pancreatic Cancer: A Comprehensive Systematic Review
Source: Int J Mol Sci. 2025 Mar 14;26(6):2620. doi: 10.3390/ijms26062620 (PMC11942318; doi:10.3390/ijms26062620)
Supplement: Supplementary file 1 [file ijms-26-02620-s001.zip › supplementary_materials.pdf]

SUPPLEMENTARY MATERIALS

Table S1. Search strategy.

PubMed	(“Pancreatic Neoplasms”[MeSH Terms])<br>AND<br>(“Immunotherapy”[MeSH Terms] OR “immune checkpoint inhibitor*”[Title/Abstract] OR “immune checkpoint blocker*”[Title/Abstract] OR “CTLA 4 Inhibitor*”[Title/Abstract] OR “Cytotoxic T Lymphocyte Associated Protein 4 Inhibitor*”[Title/Abstract] OR “PD 1 Inhibitor*”[Title/Abstract] OR “Programmed Cell Death Protein 1 Inhibitor*”[Title/Abstract])
Embase	('pancreas tumor'/mj OR 'pancreas cancer'/mj OR 'pancreas carcinoma'/mj)<br>AND<br>('immunotherapy'/mj) OR ((immune NEXT/1 checkpoint NEXT/1 inhibitor*):ti,ab,kw) OR ((immune NEXT/1 checkpoint NEXT/1 blocker*):ti,ab,kw) OR ((ctla NEXT/1 4 NEXT/1 inhibitor*):ti,ab,kw) OR ((cytotoxic NEXT/1 t NEXT/1 lymphocyte NEXT/1 associated NEXT/1 protein NEXT/1 4 NEXT/1 inhibitor*):ti,ab,kw) OR ((pd NEXT/1 1 NEXT/1 inhibitor*):ti,ab,kw) OR ((programmed NEXT/1 cell NEXT/1 death NEXT/1 protein NEXT/1 1 NEXT/1 inhibitor*):ti,ab,kw)
Central	1. MeSH descriptor: [Pancreatic Neoplasms] explode all trees<br>2. MeSH descriptor: [Immunotherapy] explode all trees<br>3. immune NEXT/1 checkpoint NEXT/1 inhibitor* OR immune NEXT/1 checkpoint NEXT/1 blocker* OR ctla NEXT/1 4 NEXT/1 inhibitor* OR cytotoxic NEXT/1 t NEXT/1 lymphocyte NEXT/1 associated NEXT/1 protein NEXT/1 4 NEXT/1 inhibitor* OR pd NEXT/1 1 NEXT/1 inhibitor* OR programmed NEXT/1 cell NEXT/1 death NEXT/1 protein NEXT/1 1 NEXT/1 inhibitor*<br>4. #2 OR #3<br>5. #1 AND #4
Scopus	("Pancreas carcinoma*" OR "Pancreas neoplasm*" OR "Pancreas Cancer*" OR "Pancreatic Carcinoma*" OR "Pancreatic neoplasm*" OR "Pancreatic Cancer*" OR "neoplasia pancreas" OR "neoplastic pancreas" OR "neoplastic pancreatic" OR "pancreas tumorigenesis" OR "pancreatic tumorigenesis" OR "pancreas tumor*" OR "pancreas tumour*" OR "pancreatic tumor*" OR "pancreatic tumour*")<br>AND<br>(“immune checkpoint inhibitor*” OR “immune checkpoint blocker*” OR “CTLA 4 Inhibitor*” OR “Cytotoxic T Lymphocyte Associated Protein 4 Inhibitor*” OR “PD 1 Inhibitor*” OR “Programmed Cell Death Protein 1 Inhibitor*”)

Figure S1. Baujat plot for OS (Overall Survival) and PFS (Progression Free Survival).

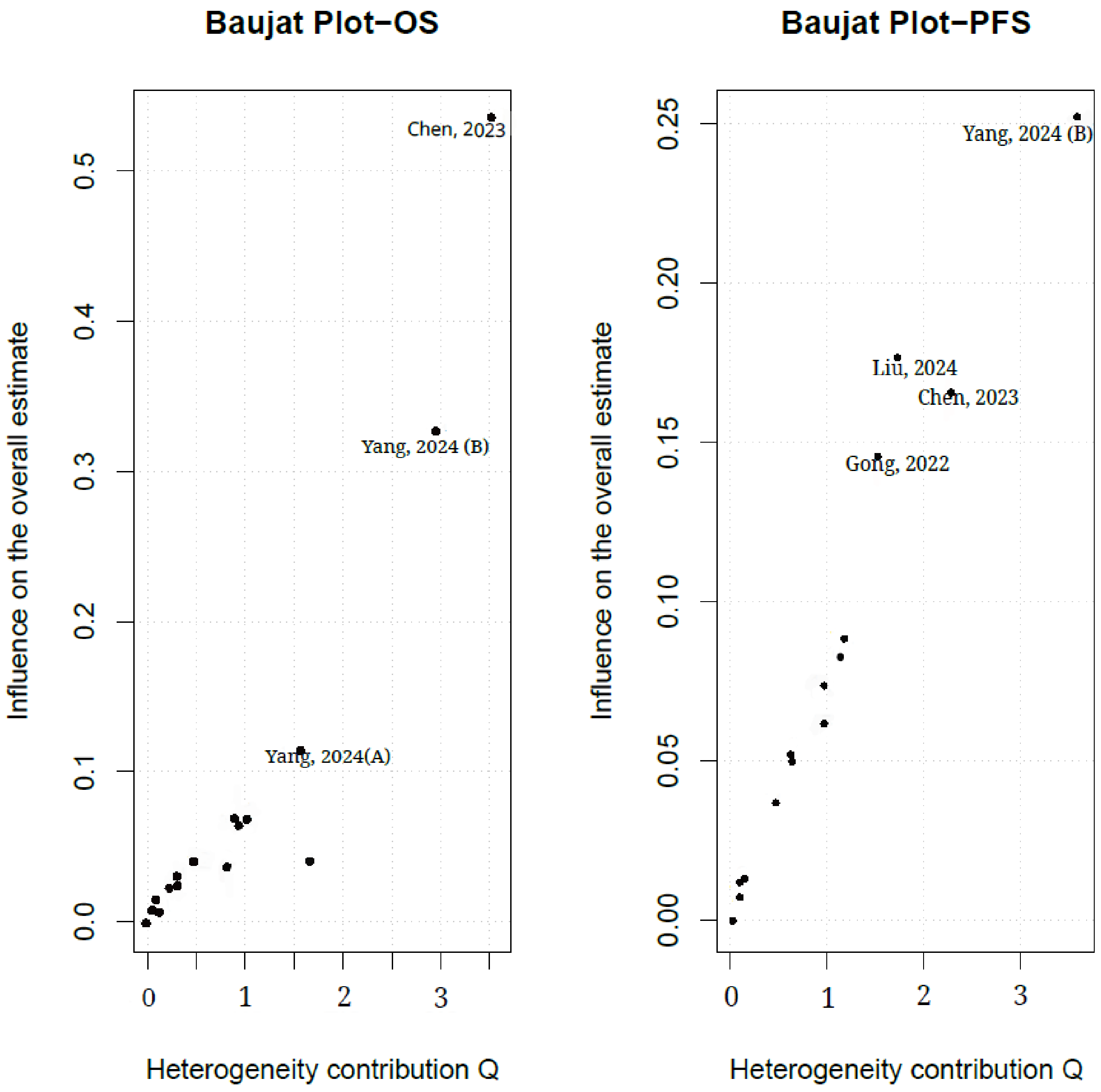

Table S2. NIH Quality Assessment Tool Evaluation Summary.

Study Name	Clarity of Objectives	Selection of Participants	Outcome Measurement	Data Completeness	Overall Rating
André 2023	Good	Good	Good	Fair	Good
Bockorny 2020	Good	Fair	Good	Fair	Fair
Callahan 2024	Good	Fair	Good	Fair	Fair
Hong 2022	Good	Good	Good	Good	Good
Lemech 2023	Good	Fair	Good	Fair	Fair
Marabelle 2020	Good	Good	Good	Good	Good
Melisi 2021	Good	Good	Good	Fair	Good
Parikh 2021	Good	Fair	Good	Fair	Fair
Reiss 2022	Good	Good	Good	Good	Good
Renouf 2022	Good	Good	Good	Fair	Good
Royal 2010	Good	Fair	Good	Fair	Fair
Tsujikawa 2020	Good	Good	Good	Good	Good
Wang 2024	Good	Good	Good	Good	Good
Xie 2020	Good	Good	Good	Fair	Fair
Curigliano 2021	Good	Good	Good	Fair	Fair
Hedge 2021	Good	Good	Good	Fair	Fair
Kitano 2020	Good	Good	Good	Fair	Fair
Muik 2020	Good	Good	Good	Fair	Fair
Naing 2023	Good	Good	Good	Fair	Fair
Papadopoulos 2023	Good	Good	Good	Fair	Fair
Voisin 2020	Good	Good	Good	Fair	Fair
Yamamoto 2024	Good	Good	Good	Fair	Fair
Zamarin 2020	Good	Good	Good	Fair	Fair
Zheng 2021	Good	Good	Good	Fair	Fair

Table S3. Newcastle-Ottawa Scale (NOS) Assessment Summary

Study Name	Selection (0-4)	Comparability (0-2)	Outcome (0-3)	Total Score (0-9)
Chen 2023	4	2	3	9
Cheng 2023	3	1	3	7
Gong 2022	4	2	2	8
Liu 2022	4	2	3	9
Liu 2024	4	2	3	9
Ma 2020	4	1	3	8
Sun 2018	4	1	3	8
Taieb 2023	4	2	3	9
Yang 2024	3	2	3	8
Zhang 2022	4	2	3	9
